# Distinct patterns of hybridization across a suture zone in a coral reef fish (*Dascyllus trimaculatus*)

**DOI:** 10.1002/ece3.6068

**Published:** 2020-02-27

**Authors:** Eva Salas, Jean‐Paul A. Hobbs, Moisés A. Bernal, W. Brian Simison, Michael L. Berumen, Giacomo Bernardi, Luiz A. Rocha

**Affiliations:** ^1^ Ichthyology Department California Academy of Sciences San Francisco CA USA; ^2^ Ecology and Evolutionary Biology Department University of California Santa Cruz Santa Cruz CA USA; ^3^ School of Biological Sciences The University of Queensland Brisbane QLD Australia; ^4^ Department of Biological Sciences Auburn University Auburn AL USA; ^5^ Center for Comparative Genomics California Academy of Sciences San Francisco CA USA; ^6^ Red Sea Research Center, Biological and Environmental Science and Engineering Division King Abdullah University of Science and Technology (KAUST) Thuwal Saudi Arabia

**Keywords:** *Dascyllus trimaculatus*, hybrid zones, Indo‐Pacific, introgression, mitochondrial DNA, phylogeography, RADSeq

## Abstract

Hybrid zones are natural laboratories for investigating the dynamics of gene flow, reproductive isolation, and speciation. A predominant marine hybrid (or suture) zone encompasses Christmas Island (CHR) and Cocos (Keeling) Islands (CKE), where 15 different instances of interbreeding between closely related species from Indian and Pacific Oceans have been documented. Here, we report a case of hybridization between genetically differentiated Pacific and Indian Ocean lineages of the three‐spot dascyllus, *Dascyllus trimaculatus* (Rüppell, 1829). Field observations indicate there are subtle color differences between Pacific and Indian Ocean lineages. Most importantly, population densities of color morphs and genetic analyses (mitochondrial DNA and SNPs obtained via RADSeq) suggest that the pattern of hybridization within the suture zone is not homogeneous. At CHR, both color morphs were present, mitochondrial haplotypes of both lineages were observed, and SNP analyses revealed both pure and hybrid genotypes. Meanwhile, in CKE, the Indian Ocean color morphs were prevalent, only Indian Ocean mitochondrial haplotypes were observed, and SNP analysis showed hybrid individuals with a large proportion (~80%) of their genotypes assigning to the Indian Ocean lineage. We conclude that CHR populations are currently receiving an influx of individuals from both ocean basins, with a greater influence from the Pacific Ocean. In contrast, geographically isolated CKE populations appear to be self‐recruiting and with more influx of individuals from the Indian Ocean. Our research highlights how patterns of hybridization can be different at scales of hundreds of kilometers, due to geographic isolation and the history of interbreeding between lineages.

## Graphical abstract

Here, we report a case of cryptic hybridization between genetically differentiated Pacific and Indian Ocean lineages of the threespot dascyllus, *Dascyllus trimaculatus*, which overlap in the Christmas–Cocos marine suture zone. Population densities of these lineages and genetic analyses (mitochondrial DNA and SNPs) suggest that the pattern of hybridization within the suture zone is not homogeneous: At Christmas Island, hybridization is ongoing; whereas at Cocos (Keeling) Islands, the lineages underwent past hybridization events that resulted in genetically stable allelic frequencies. We conclude that Cocos Island populations are geographically isolated and are self‐recruiting. In contrast, Christmas populations are currently connected and receiving constant influx from both lineages, with major influence from the Pacific Ocean.

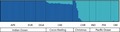

## 1 INTRODUCTION

Hybridization, or the interbreeding between species or subpopulations, is common in areas where allopatric lineages overlap (Hewitt, 1988). Hybrid zones located at the edge of biogeographic provinces (i.e., suture zones) are areas where regional biotas interact, promoting reproduction between closely related groups (Remington, 1968). These areas are widely recognized as natural laboratories for investigating the dynamics of gene flow, reproductive isolation, and speciation (Hewitt, 1988; Remington, 1968). Traditionally, hybridization has been associated with hampering differentiation, by allowing the exchange of genetic material (Abbott et al., 2013), which can ultimately lead to the fusion of divergent lineages (Rudman & Schluter, 2016). Hybridization has also been associated with the production of maladapted individuals, leading to evolutionary dead‐ends (Barton, 2001). However, more recent studies suggest this process can also have beneficial outcomes, as advantageous traits can be shared between multiple groups (Pardo‐Diaz et al., 2012; Runemark, Vallejo‐Marin, & Meier, 2019), hybrids have higher fitness than their parental lineages (i.e., hybrid vigor; Chen, 2013), and hybridization can also lead to the formation of new species and generate adaptive radiations (Meier, Marques, Wagner, Excoffier, & Seehausen, 2018; Meier et al., 2019; Seehausen, 2004).

Until recently, hybridization was considered relatively rare in marine environments (Arnold, 1997). However, ongoing ecological and molecular research in marine suture zones has led to a stark increase in the number of confirmed cases of hybridization (DiBattista et al., 2015; He et al., 2019; Hobbs & Allen, 2014; Hobbs, van Herwerden, Pratchett, & Allen, 2014; Montanari, Hobbs, Pratchett, & van Herwerden, 2016). To date, two major marine suture zones have been recognized for coral reef fishes: the well‐studied zone that encompasses Christmas Island (CHR) and Cocos (Keeling) Islands (CKE) in the eastern Indian Ocean (Hobbs & Allen, 2014; Hobbs, Frisch, Allen, & Van Herwerden, 2008), and the less explored zone in the Socotra Archipelago (DiBattista et al., 2015). In the former, glaciations led to sea level drops that exposed the Sunda Shelf, restricting water (and consequently gene flow) between the tropical Indian and Pacific Oceans, which resulted in divergence of many marine populations and species (Barber, Palumbi, Erdmann, & Moosa, 2000; Gaither & Rocha, 2013; Ludt & Rocha, 2015). Since the end of the last ice age, sea levels rose and the previously exposed land masses are now shallow reef habitat, allowing the admixture of Indian and Pacific Ocean lineages.

To date, 15 cases of hybridization have been observed in the Cocos–Christmas suture zone, involving 27 species across eight families (Hobbs & Allen, 2014). These cases commonly follow Hubbs' principle, which takes place when one or both parental species exhibit low frequencies (DiBattista et al., 2015; Hobbs et al., 2008; Hubbs, 1955). Previous studies suggest a potential bias when reporting these instances, as most cases belong to sister species that produce hybrids with a clear intermediate coloration, such as butterflyfishes (Chaetodontidae; Hobbs & Allen, 2014). Further, 14 out of those 15 hybrid cases are from broadcast spawning species, the other case is for substrate spawners *Chromis fieldi* and *C. margaritifer* (He et al., 2019). Damselfishes (family Pomacentridae) are one of the most diverse families of coral reef fishes, and hybridization events are not uncommon (i.e., anemonefishes, Litsios & Salamin, 2014). However, relatively few hybrids have been reported in the Cocos–Christmas suture zone, raising the issue of potential cryptic hybridization. Cryptic hybrids are common in the marine environment (Richards & Hobbs, 2015), and they could represent a significant portion of the cases of hybridization in the Cocos–Christmas suture zone (Hobbs & Allen, 2014).

The three‐spot dascyllus (*Dascyllus trimaculatus,* (Rüppell 1829) species complex offers interesting opportunities for understanding the dynamics of speciation and introgression. They can be split into five major lineages (some with taxonomic recognition) based on well‐established patterns of genetic divergence and biogeography: (a) the Marquesas endemic *D. strasburgi*, (b) the Hawaiian and Johnston Atoll *D. albisella*, (c) the French Polynesian *D. trimaculatus,* (d) the Pacific rim lineage comprising two introgressed groups: *D. trimaculatus* and *D. auripinnis*, and (e) the Indian Ocean *D. trimaculatus* (Bernardi, Holbrook, & Schmitt, 2001; Bernardi, Holbrook, Schmitt, & Crane, 2003; Bernardi, Holbrook, Schmitt, Crane, & DeMartini, 2002; Leray et al., 2010; McCafferty et al., 2002). A widespread population genetic study of this group suggested a combination of historical and ecological factors promoted the divergence of this complex (Leray et al., 2010). The present study aims to elucidate the genomic divergence of the Pacific and Indian Ocean lineages of *D. trimaculatus* (isolated by limited water circulation in the Sunda Shelf during the Pleistocene; Leray et al., 2010)*,* and their interaction in the Cocos–Christmas suture zone using a combination of genomic and field approaches. We hypothesize that hybridization is occurring between these lineages in the suture zone and that the genomic structure of hybrids will be different for the islands due to their geographic positioning. As CHR is closer to the Pacific Ocean and CKE is closer to the Indian Ocean, our expectation is that hybrid individuals from CHR will have a higher proportion of Pacific ancestry, and conversely, CKE hybrid individuals will have a higher proportion of Indian ancestry. Our study provides an example on the importance of suture zones for genomic exchange between closely related lineages, while highlighting how dynamics of hybridization can be different at small geographic scales (hundreds of kilometers).

## 2 MATERIALS AND METHODS

The species *D. trimaculatus* is a planktivore commonly found in lagoons and reef walls of the Indo‐Pacific (Bernardi et al., 2001; Randall & Allen, 1977). They are sexually monomorphic and display complex reproductive behavior where the males attract females with acoustic signals and movements (Fishelson, 1998). Eggs are demersal and the pelagic larval duration is estimated to be 22–26 days (Wellington & Victor, 1989). Juveniles are associated with anemones, urchins, or branching corals. As adults, they shift away from these associations, but typically remain nearby (Randall & Allen, 1977).

### 2.1 Sampling

**Figure 1:**
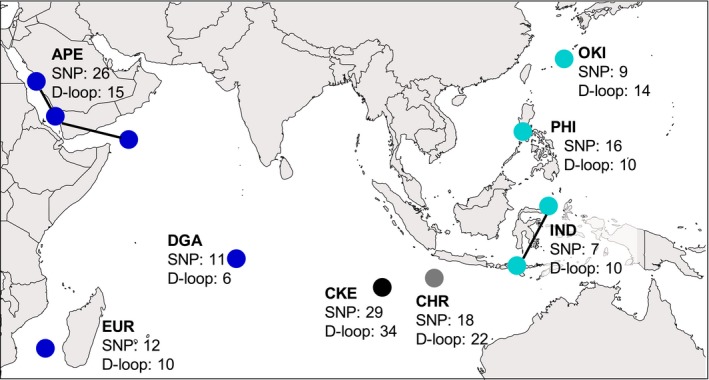
Collection sites and sample sizes for the SNP and the mitochondrial (D‐loop) dataset of *Dascyllus trimaculatus*. Dark blue circles indicate Indian Ocean populations: APE: Arabian Peninsula; EUR: Europa, Scattered Islands; DGA: Diego Garcia, Chagos Archipelago; black and gray circles indicate hybrid zone populations: CKE: Cocos (Keeling) Islands; CHR: Christmas Island; and light blue circles indicate Pacific populations: IND: Indonesia; PHI: Philippines; OKI: Okinawa. The SNP samples of APE (*n* = 26) were a combination of three locations, indicated by the circles joined by lines: Thuwal *n* = 21, Farasan Islands *n* = 2, and Socotra *n* = 3. The samples of IND (*n* = 7) were also a combination of two locations: Manado *n* = 3 and Komodo *n* = 4

Previous studies have indicated significant genetic differences between the Indian and Pacific lineages of *D. trimaculatus* (Leray et al., 2010). Hence, the sampling design aimed to gather individuals from the suture zone and locations representing pure Pacific or pure Indian Ocean lineages (Figure 1). The Indian Ocean lineage was represented by samples of the Arabian Peninsula (APE), the western Indian Ocean (Europa; EUR), and the central Indian Ocean (Diego de Garcia; DGA). The Pacific Ocean lineage was represented by samples from Indonesia (IND), the Philippines (PHI), and Okinawa, Japan (OKI). Fish were collected between 2010 and 2014, and the samples from IND and three from CKE were part of an earlier study (Leray et al., 2010). All experiments were performed in accordance with UCSC Institutional Animal Care and Use Committee (IACUC/BERNG‐1601).

### 2.2 Color morphs and visual surveys

**Figure 2:**
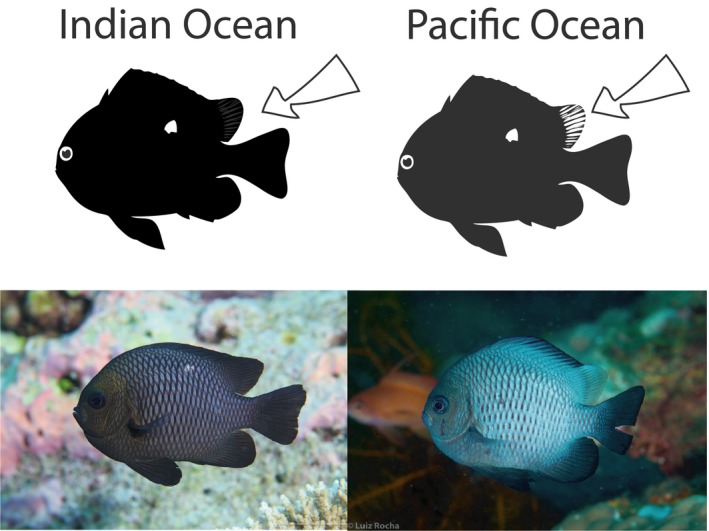
The Indian and Pacific *Dascyllus trimaculatus* are distinguished by the trailing edge of the dorsal fin. While the coloration in the body is variable, the coloration of the fin does not change. Image on the left corresponds to a fish from the Maldives (photograph by Tane Sinclair‐Taylor), and the one of the right is from the Philippines (photograph by Luiz A. Rocha)

**Figure 3:**
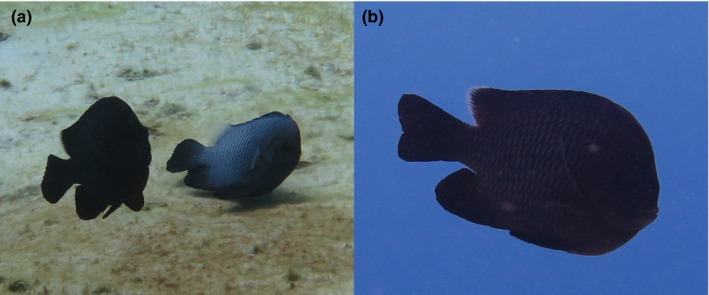
Mixed morphotypes mating and intermediate morph of *Dascyllus trimaculatus*. (a) Christmas Island is one of the few places where Pacific and Indian morphs of *D. trimaculatus* overlap and interbreed. (b) We identified fish with various ranges of intermediate coloration on the trailing edge of the dorsal fin, but the intermediate coloration is very subtle ( photographs by: Jean‐Paul A. Hobbs)

We explored whether there were consistent differences in color between Pacific and Indian Ocean lineages across the species' distribution range using personal field observations and photographs. In addition, 43 georeferenced images and videos from Flickr (http://flickr.com) were analyzed (24 Indian Ocean and 19 Pacific Ocean locations). Individuals were categorized into three phenotypes based on color patterns: (a) black rear end of the dorsal fin, (b) clear rear end of the dorsal fin, and (c) intermediate forms with partially black and transparent dorsal fins (Figures 2 and 3).

The abundance and color morph frequencies of *D. trimaculatus* at CHR and CKE were estimated using standard underwater visual census methods. Three replicate 50 × 5 m belt transects were conducted at two depths (5 and 20 m) across eight sites at CHR and at seven sites at CKE.

### 2.3 Mitochondrial DNA sequencing and data analysis

Genomic DNA was extracted using the Qiagen DNeasy animal blood and tissue kit (Qiagen). A 495 base pair fragment of the mitochondrial control region (D‐loop) was amplified in 121 individuals using CR‐A and CR‐E primers (Lee, Conroy, Howell, & Kocher, 1995). PCRs (10 μl) were performed using 5 μl of multiplex PCR mix (Qiagen), 0.5 μl of CRA 10 μM, 0.5 μl of CRE 10 μM, 3 μl of water, 1 μl of DNA (30–100 ng/μl). Touchdown PCRs were set up as follows: 15 min at 95°C, followed by 20 cycles of 30 s at 95°C, 60 s at 58°C, 90 s at 72°C. During these cycles, the temperature was decreased −0.4°C every minute. This was followed by 15 cycles of 30 s at 95°C, 60 s at 50°C, 90 s at 72°C; and a final extension of 72°C for 10 min. DNA was purified using exonuclease I and FastAP™ thermosensitive alkaline phosphatase (ExoFAP; USB), running it for 60 min at 37°C, followed by 15 min at 85°C. DNA was sequenced in the forward and reverse direction using fluorescent‐labeled dye (BigDye 3.1, Applied Biosystems Inc.) using an ABI 3730xl Genetic Analyzer. Sequences were aligned and trimmed using Geneious 6.06 (Kearse et al., 2012), and final edits were performed manually.

In total, 121 individuals from eight populations (Figure 1) were used in the mitochondrial DNA data analysis. The haplotype and nucleotide diversity of each lineage and of the populations in the suture zone was estimated with arlequin 3.5.1.2 (Excoffier & Lischer, 2010). In order to assess genetic divergence between pure lineages and hybrid sites, estimates of pairwise Phi‐st (*Φ*_ST_) were calculated with the same software. For the above analysis, we pooled the population samples of each pure lineage in the Indian Ocean (APE, EUR, DGA) and Pacific (IND, PHI, OKI; see Table 1). Significance of *Φ*_ST_ values was tested with 10,000 permutations. The Kimura 2 Parameter was selected as the best model of sequence evolution in jmodeltest2 (Darriba, Taboada, Doallo, & Posada, 2012). Sequential Bonferroni corrections were applied for multiple comparisons (Rice, 1989).

**Table 1:** *Dascyllus trimaculatus* SNP *F*_ST_ (bottom) and mitochondrial *Φ*_ST_ (top). All comparisons were significant after sequential Bonferroni corrections, and all comparisons had *p* < .001. Indian Ocean populations include APE: Arabian Peninsula; EUR: Europa, Scattered Islands; DGA: Diego Garcia, Chagos Archipelago. Pacific populations include IND: Indonesia; PHI: Philippines; OKI: Okinawa. For SNP data: Indian *n* = 49, Cocos (Keeling) *n* = 29, Christmas *n* = 18, Pacific *n* = 32. For mitochondrial data: Indian *n* = 31, CKE *n* = 34, CHR *n* = 22, Pacific *n* = 34

	Indian	Cocos	Christmas	Pacific
Indian	—	0.0787	0.3822	0.7133
Cocos	0.0241	—	0.4745	0.7855
Christmas	0.1284	0.0815	—	0.1161
Pacific	0.2526	0.1827	0.0330	—

In order to assign each individual haplotype from the hybrid zone to the Indian and Pacific lineages, a minimum spanning haplotype network was constructed using the software popart (Leigh & Bryant, 2015).

### 2.4 RADSeq library preparation, sequencing, and assembly

Genomic analyses were done on single nucleotide polymorphisms (SNPs), obtained via double‐digest restriction site‐associated DNA sequencing (ddRAD). The library preparation was performed for 224 samples for two different projects including this study. Library preparation followed the protocol described by Peterson, Weber, Kay, Fisher, and Hoekstra (2012), starting with 500 ng of total DNA per sample. Samples were digested for 3 hr at 37°C, using restriction enzymes SphI and MluCl (New England Biolabs). Digests were quantified with Qubit 2.0 Fluorometer using the HS dsDNA assay kit (Life Technologies) and then cleaned with Dynabeads/M270 Streptavidin (Life Technologies). Ligation was performed with P2 universal adaptors and 14 sets of 16 individuals were barcoded with unique P1 adaptors. After ligation, each group of 16 individuals was pooled and bead‐cleaned. Pools were size‐selected for 400 bp using a 2% agarose gel that ran for 45 min and purified with the Zymoclean Gel DNA Recovery Kit. Unique Illumina Indexes were added to each of the 14 pools and the libraries were amplified with 10 PCR cycles using the high‐fidelity Platinum Taq DNA polymerase (Thermo Fisher Scientific). The concentration of each pool was quantified using a High Sensitivity Kit on a 2100 Bioanalyzer (Agilent Technologies), then standardized, and combined into a single tube for sequencing. Samples were sequenced on two lanes of an Illumina Hi‐Seq 2000, at the KAUST Bioscience Core Laboratory, which resulted in 251,425,358 and 270,346,963 single‐end 100‐bp reads.

Sequences were processed using stacks v1.09 pipeline (Catchen, Amores, Hohenlohe, Cresko, & Postlethwait, 2011; Catchen, Hohenlohe, Bassham, Amores, & Cresko, 2013). Raw data were demultiplexed and filtered using the “process_rad_tags” script. Average quality scores were determined within a sliding window 15% the length of the sequence, and reads with any score below 90% of being correct were discarded. Sequences were trimmed to 95 bp, and loci were assembled using the stacks “de_novo_map.pl” pipeline, using a minimum of three identical reads to create a stack (*m* = 3), three mismatches allowed between loci within an individual (*M* = 3), five mismatches when aligning reads (*N* = 5), and two mismatches when building the catalog (*n* = 2). We used the “populations” script to obtain loci shared between the eight populations (*p* = 8), in at least 65% of individuals within a population (*r* = 0.65) and with coverage of 8× (*m* = 8). We used only the first SNP of each sequence and removed loci with minor allele frequencies lower than 5%. The filtering resulted in a total of 128 individuals with 2,818 loci, with 83% completeness (see Figure 1 for samples per site). For all downstream analyses, we used the structure output file produced by stacks which was converted to other file formats using pgdspider 2.0 (Lischer & Excoffier, 2012).

### 2.5 Analysis of RADSeq data

A total of individuals from the eight populations mentioned above (Figure 1) were used in the RADSeq data analysis, most of these were also part of the mtDNA analyses. The samples of APE (*n* = 26) were a combination of three locations: Thuwal *n* = 21, Farasan Islands *n* = 2, and Socotra *n* = 3 (Figure 1). The samples of IND (*n* = 7) were a combination of two locations: Manado *n* = 3 and Komodo *n* = 4 (Figure 1). No significant genetic differences were observed between these localities, so they were combined into regional pools.

To quantify the extent of genetic differentiation between lineages and the hybrid zone, pairwise *F*_ST_ values were estimated between CHR, CKE, and the Pacific/Indian Ocean lineages using arlequin with 10,000 permutations. For this analysis, we pooled the population samples of each pure lineage in the Indian Ocean (APE, EUR, DGA) and Pacific (IND, PHI, OKI; see Table 1). In addition, pairwise *F*_ST_ was calculated per population separately. Sequential Bonferroni corrections were applied for multiple comparisons (Rice, 1989).

We examined how the distribution of *F*_ST_ per locus varies when comparing lineages and the suture zone. We compared the Indian lineage (APE, EUR, DGA) versus CHR and versus CKE; the Pacific lineage (IND, PHI, OKI) versus CHR and versus CKE; the Pacific versus the Indian lineage, and last, we compared the suture zone populations (CHR vs. CKE). These results are presented in a histogram prepared in R, using ggplot2 (Wickham, 2016). Further, we made comparisons of the distribution and average pairwise *F*_ST_ values within Indian Ocean populations (APE vs. EUR, APE vs. DGA, EUR vs. DGA), within Pacific Ocean populations (IND vs. PHI, IND vs. OKI, PHI vs. OKI), between the Indian Ocean lineage (APE, EUR, DGA, pooled) and the suture zone populations (CHR and CKE, pooled), and between the Pacific Ocean lineage (IND, PHI, OKI, pooled) and the suture zone (CHR and CKE, pooled). These results are presented in a boxplot prepared in R, using ggplot2. The *F*_ST_ values per locus were obtained with the PEGAS R package (Paradis, 2010).

To further investigate population structure, genetic assignment of individuals of each population was calculated with structure (Pritchard, Stephens, & Donnelly, 2000) using correlated allele frequencies in an admixture model, for 1 million Markov chain Monte Carlo (MCMC) iterations and 10% burn‐in. We ran 10 simulations for each group (*K* = 1 to *K* = 9). The most likely number of clusters (*K*) was determined with the Evanno method (Evanno, Regnaut, & Goudet, 2005) using structure harvester (Earl & vonHoldt, 2012). Results of the 10 simulations of the most likely K were aligned using clumpp (Jakobsson & Rosenberg, 2007), and later graphed with distruct (Rosenberg, 2004).

In addition, to visualize the relationship between populations in the suture zone and the Indian and Pacific lineages, we ran a discriminant analysis of principal components (DAPC; Jombart, Devillard, & Balloux, 2010). DAPC describes genetic clusters using a multivariate method that combines discriminant analysis (DA) with principal component analysis (PCA) to best summarize differences between groups (populations) while minimizing variation within groups. DAPC also provides the probability of assignment of individuals to specific groups based on the retained discriminant functions, which can be interpreted as the proximity of individuals to the different clusters (Jombart et al., 2010). The analysis was executed using adegenet (Jombart, 2008) for R (R Development Core Team, 2015), using the best number of principal components (PCs) identified with the cross‐validation method (*xValDapc* function). To investigate the putative number of clusters (groups) in the data, we applied the “find.clusters” algorithm, which finds the number of groups maximizing the variation between groups, and compares different clustering solutions using Bayesian information criterion (BIC, Jombart et al., 2010). The DAPC was generated using the number of PCs and clusters identified.

To investigate if the structure signal is driven by SNPs highly differentiated between Pacific and Indian Ocean populations, we ran additional structure analysis using a subset of neutral loci and another subset of highly differentiated loci. To classify loci, we ran the *F*_ST_ outlier approach implemented in arlequin. The method consists of a modified fdist procedure under a hierarchical island model that simulates a null distribution across loci as a function of heterozygosity and determines outliers as those that show either significantly higher or lower *F*_ST_ (Excoffier, Hoffer, & Foll, 2009). We ran 50,000 simulations with 100 demes per group, with minimum and maximum expected heterozygosities of 0 and 0.5, respectively. Based on the results of this analysis, we classified each locus into one of three categories: neutral loci (between the 95% quantiles), balancing loci outliers (below the 1% quantile), or divergent loci outliers (above the 99% quantile).

In addition, we used this classification to compare allele frequencies across the sampled populations. We calculated the allelic frequencies of each locus with arlequin, and results were plotted with ggplot2 in R.

In order to identify fixed loci between Pacific and Indian Ocean populations, we grouped all individuals from the Indian Ocean into a single population (APE, EUR, DGA) and did the same for all samples from the Pacific Ocean (IND, PHI, OKI). Samples from the suture zone of CHR and CKE were excluded from this analysis. We used the “populations” script to identify loci shared between the Pacific and Indian ocean populations (*p* = 2), in at least 65% of individuals within a population (*r* = 0.65). We used only the first SNP for each locus, estimated the pairwise *F*_ST_ between the groups and only kept fixed loci, resulting in 50 loci with *F*_ST_ = 1. We generated a whitelist of all 50 fixed loci to run a population script on four populations (Pacific, Indian Ocean, CHR, and CKE). We then generated a genepop file, to obtain the allelic frequencies of the 50 fixed loci among the Pacific and Indian ocean lineages, as well as for the two populations in the suture zone (CHR and CKE). Of those 50 loci, 5 were not found in the suture zone populations. We used the remaining 45 loci to estimate the average allelic frequencies between the Indian Ocean, Pacific Ocean, CHR, and CKE.

## 3 RESULTS

### 3.1 Color morph and visual survey results

The photographs obtained in the field, surveys, and georeferenced images/videos from Flickr used in this study revealed consistent patterns in the color of the trailing edge of the dorsal fin of *D. trimaculatus*. All of the observed individuals that live in the Indian Ocean (outside of the suture zone) show a black trailing edge of the dorsal fin, whereas all of the Pacific Ocean individuals have a transparent/white trailing edge (Figure 2, Table A1). This phenotypic difference between Pacific and Indian Ocean *Dascyllus trimaculatus* has been discussed anecdotally by naturalists before, but it was never tested in multiple locations, nor was it mentioned in the species description or in any peer‐reviewed publication. Taken together, all the data suggest that the Pacific rim lineage is morphologically different from the individuals of the Indian Ocean lineage. Even though the coloration patterns may break down in the suture zone, the trailing edge of the dorsal fin can still reveal useful information about how hybridization dynamics can affect the phenotype.

The underwater surveys of *D. trimaculatus* reveal that both color morphs co‐occur at CHR and CKE. Further, intermediate phenotypes where the trailing edge of the dorsal fin was only partially transparent and/or white were observed in the suture zone (Figure 3). At CHR, clear fin individuals were the most common phenotype and their density (0.961 per 250m^2^ ± 0.29 *SE*) was approximately double that of black fin individuals (0.451 per 250m^2^ ± 0.15 *SE*). In contrast, at CKE, black fin individuals (0.381 per 250m^2^ ± 0.14 *SE*) were the most common phenotype, and clear fin individuals were rare (0 per 250 m^2^); they were only observed sporadically outside the transects. Individuals with half clear/black were rare at both CHR (0.098 per 250m2 ± 0.07 *SE*) and CKE (0 per 250 m^2^, and a few observations outside transects).

In the hybrid zone, lineage and color do not always match. In this study, we were limited as only in a few instances we had both genetic and morphotype information. At CHR, we observed one individual (out of 4) with a full genetic color mismatch (with Pacific mtDNA haplotype, Pacific SNP genotype, and Indian color morph), and another one with only a mitochondrial mismatch (Pacific mtDNA haplotype, >80% Indian SNP genotype and Indian color morph). Five additional individuals lacked either the mtDNA data or the SNP genotype, but showed a match between color and genetics. At CKE, 24 individuals had mtDNA, SNP, and color information, and all of them had Indian mtDNA haplotype, more than 80% of the Indian SNP genotype and Indian color morph.

### 3.2 Analyses of mitochondrial DNA

**Figure 4:**
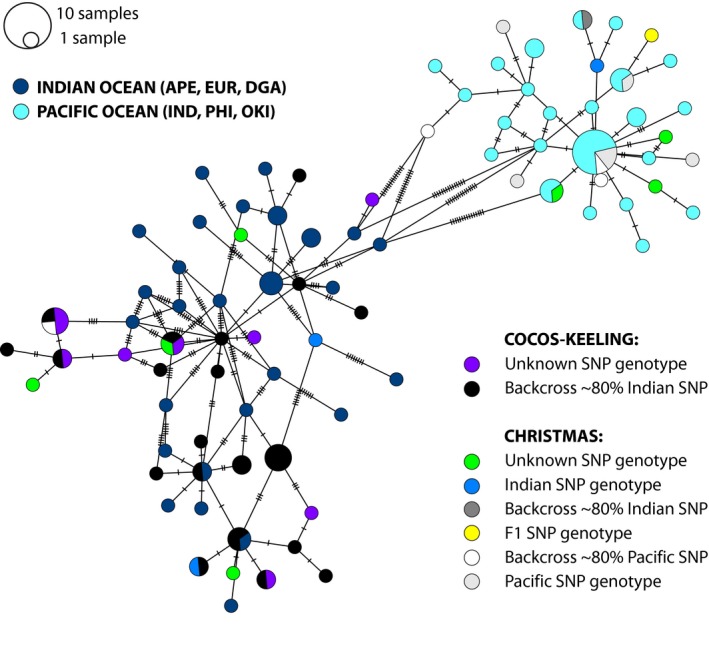
Haplotype network of *Dascyllus trimaculatus* using 495 bp of the mitochondrial D‐loop. Circles represent haplotypes; its size is proportional to the haplotype frequency. Connecting lines and each bar represent single mutation steps. Cocos (Keeling) Islands and Christmas Island individuals were additionally classified based on the SNP genotypes (if available) into: F1 hybrids, backcrosses, pure Pacific or Indian Ocean genotype

**Figure 5:**
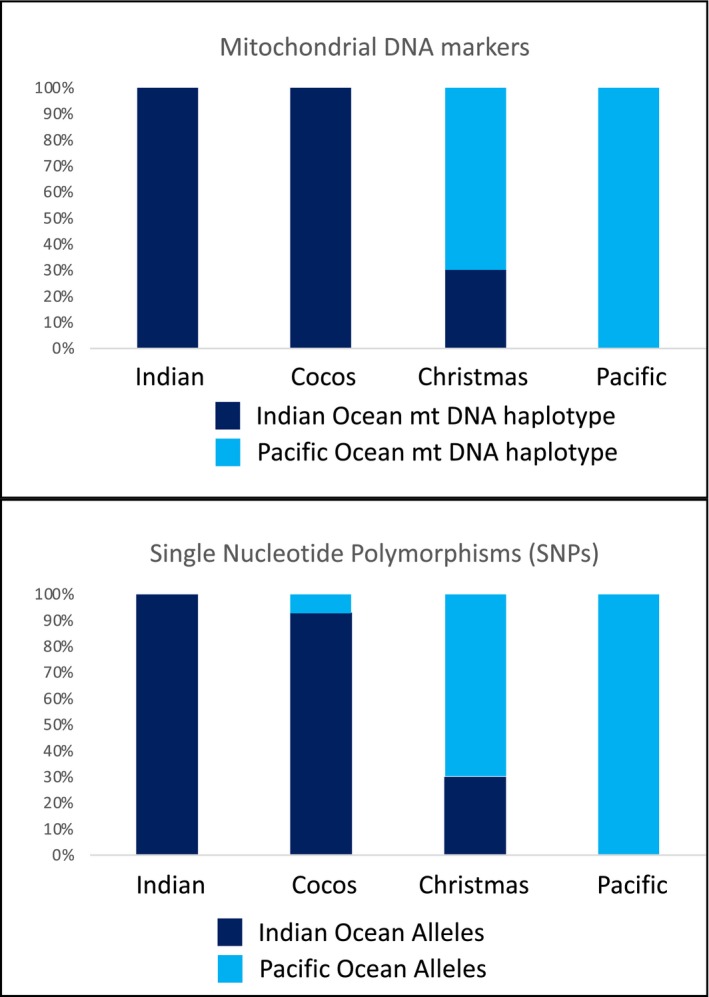
Top: Mitochondrial (mtDNA) haplotype frequency of the individuals from Cocos (Keeling) and Christmas Island. Bottom: Average allele frequencies of the 45 SNP loci that are fixed between the Indian and the Pacific lineage

**Figure A1:**
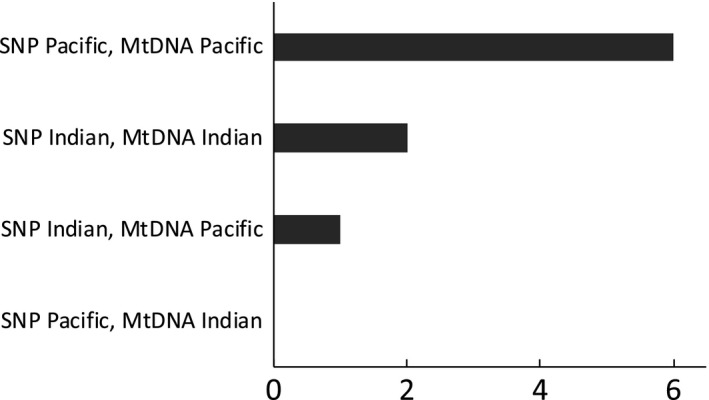
Individuals from Christmas Islands with known SNP and mitochondrial information, and that have a pure SNP genotype representative from either the Pacific or the Indian lineage.

Our analyses were consistent with previously published results that indicate a clear genetic break between Pacific and Indian Ocean populations (*Φ*_ST_ = 0.7132 between Pacific and Indian Ocean, *p* < .001, Table 1; Leray et al., 2010). In addition, significant *Φ*_ST_ values were also present when comparing Indian and Pacific Ocean populations to CHR and CKE populations (Table 1). A haplotype network shows that the CHR population comprises both Indian and Pacific haplotypes, with 7 and 15 individuals, respectively, whereas the CKE population only contains Indian Ocean haplotypes (Figures 4 and 5). A correlation between mitochondrial and nuclear origin is not always observed, as we found: an individual with pure Indian Ocean SNPs and a Pacific mitochondrial haplotype; a backcrossed individual that is assigned with 80% Indian Ocean SNPs and a Pacific mitochondrial haplotype; and a backcrossed individual assigned with 80% Pacific SNPs and an Indian Ocean mitochondrial haplotype. However, at CHR it is still more common to find ‘pure’ individuals, with a mitochondrial and nuclear origin from the same lineage (Figure A1).

Populations from pure Indian Ocean locations had higher nucleotide diversity than populations from pure Pacific locations (2.2% and 1%, respectively, Table 2). Nucleotide diversity was highest in CHR (3.9%), and even when CHR individuals with Pacific haplotypes were examined separately, these had higher nucleotide diversity than individuals from the pure Pacific locations (1.9% vs. 1%, respectively). At CKE, where only Indian haplotypes were found, nucleotide diversity was lower than in pure Indian Ocean populations (1.5% vs. 2.2%, respectively). Haplotype diversities were similar in all populations (Table 2).

**Table 2:** Haplotype and nucleotide diversity in *Dascyllus trimaculatus'* D‐loop region. Individuals from the Indian Ocean sites include APE: Arabian Peninsula; EUR: Europa, Scattered Islands; DGA: Diego Garcia, Chagos Archipelago. Individuals from the Pacific Ocean sites include IND: Indonesia; PHI: Philippines; OKI: Okinawa. Christmas Island individuals were separated per their haplotypes (Pacific or Indian), then analyzed altogether. The number of individuals used in the analysis is indicated in parenthesis

	Indian haplotype		Pacific haplotype	
	Haplotype diversity	Nucleotide diversity, PI	Haplotype diversity	Nucleotide diversity, PI
Indian sites	1 (31)	2.2% (31)	Absent	Absent
Pacific sites	Absent	Absent	0.99 (34)	1.0% (34)
Cocos (Keeling) Islands	0.99 (34)	1.5% (34)	Absent	Absent
Christmas Island	1 (7)	2.1% (7)	1 (15)	1.9% (15)
Christmas (Indian and Pacific haplotypes pooled, *n* = 22)	Haplotype diversity: 1 ± 0.0137, Nucleotide diversity 3.9% ± 2%			

### 3.3 RADSeq analyses

**Figure A2:**
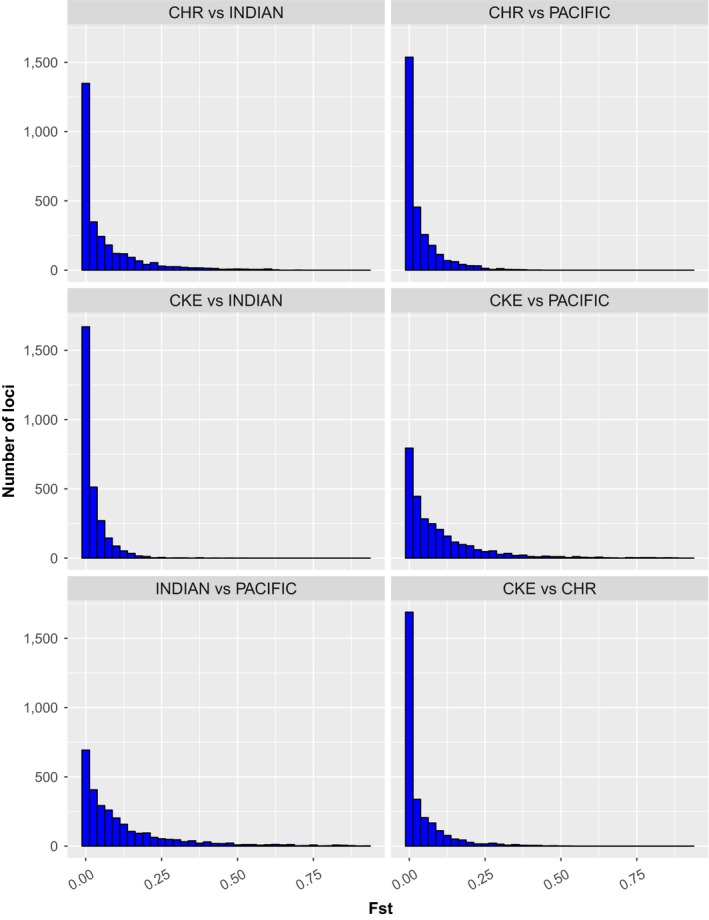
Histograms showing the distribution of locus‐specific pairwise *F* _st_ comparisons. For this graph, all negative *F* _st_ values were converted to zero. The comparisons were among the following groups: (1) Indian, a pool of APE (Arabian Peninsula), EUR (Europa, Scattered Islands), and DGA (Diego Garcia, Chagos Archipelago) populations; (2) Pacific, a pool of IND (Indonesia), PHI (Philippines) and OKI (Okinawa) populations; and the populations of the suture zone (3) CKE (Cocos(Keeling)), and (4) CHR (Christmas)

**Figure A3:**
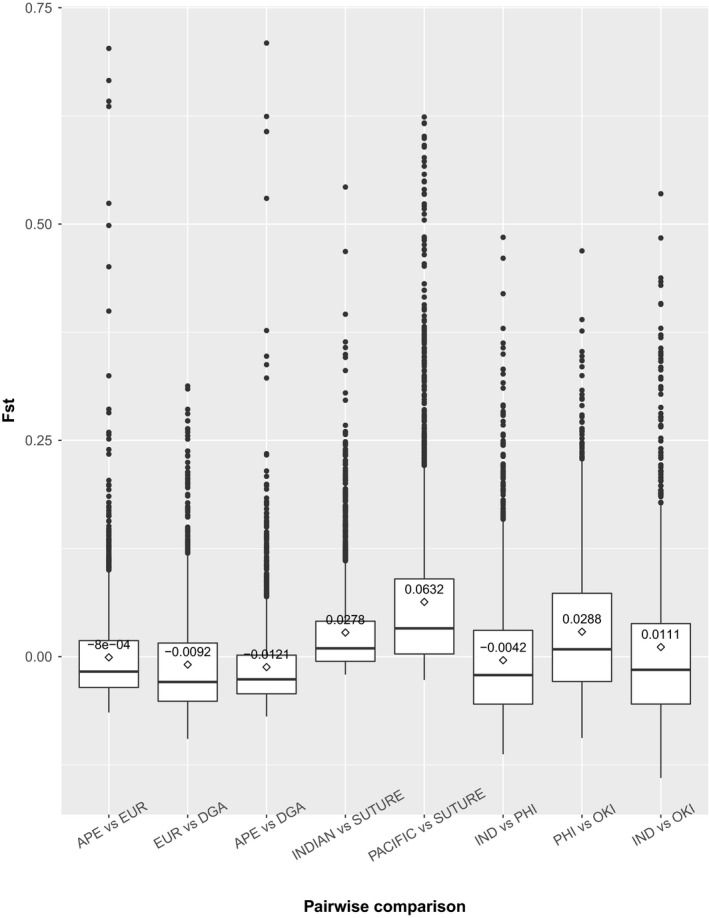
Box plot showing the distribution and average *F* _st_ values among a set of pairwise comparisons. The comparisons were within Indian Ocean populations, within Pacific Ocean populations, between the Indian Ocean lineage and the suture zone populations, and between the Pacific Ocean lineage with the suture zone populations. The Indian lineage included APE (Arabian Peninsula), EUR (Europa, Scattered Islands) and DGA (Diego Garcia, Chagos Archipelago); the suture zone included CKE (Cocos(Keeling)) and CHR (Christmas); and the Pacific lineage included IND (Indonesia), PHI (Philippines) and OKI (Okinawa)

Filtering criteria resulted in 2,818 SNP loci that were used for the genomic analyses. *F_ST_* comparisons were concordant with the results of the mtDNA analysis, as there is strong genetic differentiation between Pacific and Indian Ocean lineages (*F*_ST_ = 0.2526, *p* < .001, Table 1, Table A2). The distribution of *F*_ST_ values does not differ much when comparing CHR with either lineage, nor CHR with CKE. Meanwhile, the distribution of *F*_ST_ values shows that CKE and the Pacific lineage have more differentiated loci than when comparing CKE and the Indian lineage. (Figure A2). The suture zone appears to be more differentiated from the Pacific Ocean than the Indian Ocean lineage (Figure A3).

**Figure 6:**
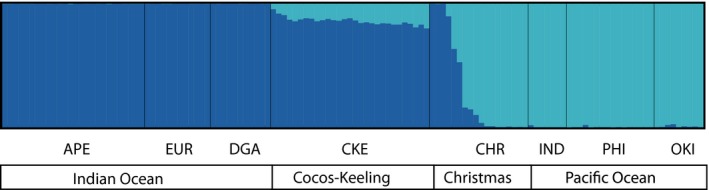
Bayesian cluster analysis performed by structure using 2,818 SNP loci of *Dascyllus trimaculatus*. The most likely number of clusters was *K = *2 (∆*K* = 14,566). The Indian Ocean populations are presented in dark blue (APE: Arabian Peninsula; EUR: Europa, Scattered Islands; DGA: Diego Garcia, Chagos Archipelago) and Pacific populations are presented in light blue (IND: Indonesia; PHI: Philippines; OKI: Okinawa). The suture zone is represented by CKE (Cocos Keeling Islands) and CHR (Christmas Island)

**Figure 7:**
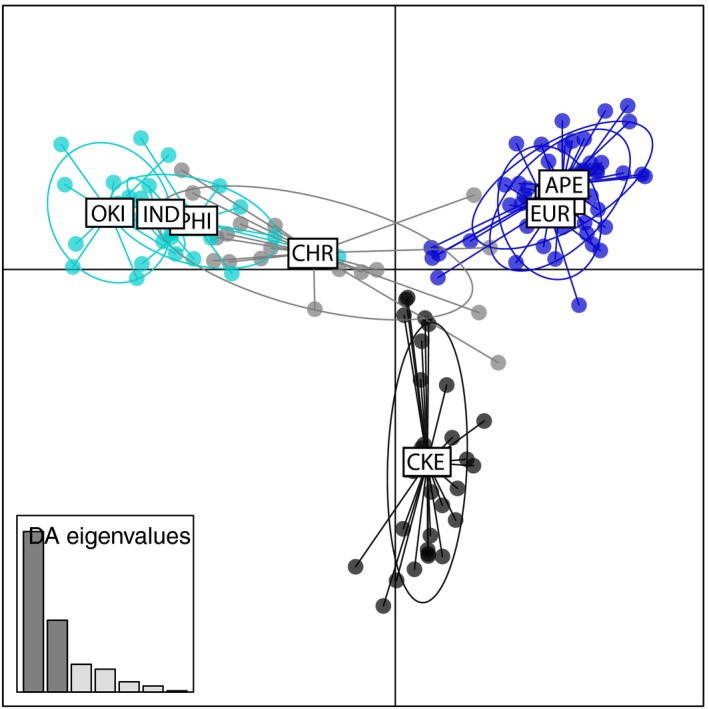
Discriminant analysis of principal components (DAPC) based on 2,818 SNP loci, showing the relationships between *Dascyllus trimaculatus* populations. Indian Ocean populations are presented in dark blue (APE: Arabian Peninsula; EUR: Europa, Scattered Islands; DGA: Diego Garcia, Chagos Archipelago), the Hybrid zone populations are presented in black and gray (CKE: Cocos Keeling Islands; CHR: Christmas Island), and Pacific populations in light blue (IND: Indonesia; PHI: Philippines; OKI: Okinawa)

**Figure A4:**
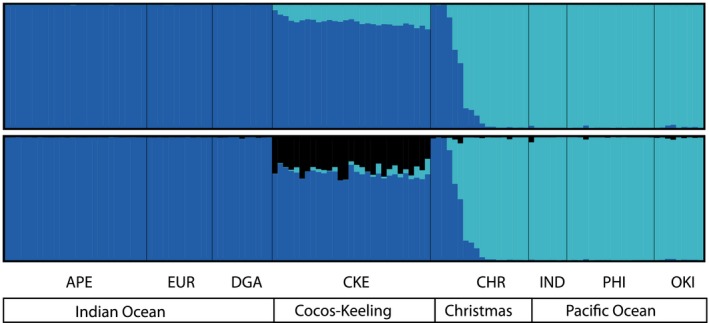
Bayesian cluster analysis performed by structure based in 2,818 SNP loci of *Dascyllus trimaculatus*. The top image shows the most likely number of clusters (*K = *2; ∆*K *= 14,566) and the bottom image shows the second most likely one (*K *= 3; ∆*K* = 489) for comparison. The locations are: APE (Arabian Peninsula), EUR (Europa, Scattered Islands), DGA (Diego Garcia, Chagos Archipelago), CKE (Cocos(Keeling)), CHR (Christmas), IND (Indonesia), PHI (Philippines) and OKI (Okinawa)

Assignment tests with structure (all 2,818 loci) showed that the most likely number of clusters is *K* = 2 (∆*K* = 14,566), and the second most likely is *K* = 3 (∆*K* = 489; Figure 6; Figure A4). The individuals from CHR consisted of both pure and hybrid genotypes, and the analysis identified F1 hybrids and admixed individuals. In CKE, no pure genotypes were found, and all the individuals were backcrosses with >80% of their genotype composition assigning to the Indian Ocean lineage (Figure 6). The DAPC analysis shows concordance with the findings of structure, as the algorithm identified the presence of two clusters, and the plot shows the separation of Pacific and Indian Ocean lineages on the primary axis of variation, with suture zone populations in between (Figure 7). The genetic signature of CKE is unique, as reflected by the characterization as a separate genetic cluster by the DAPC plot, where individuals cannot be classified within (or close to) the Pacific or Indian Ocean clusters, like the CHR individuals do. The eigenvalues of the analysis showed that 70% of the genetic structure was captured by the first two principal components (Figure 7).

**Figure A5:**
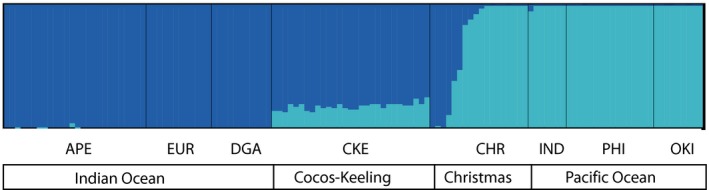
Bayesian cluster analysis performed by structure based on 1,949 neutral SNP loci of *Dascyllus trimaculatus*. The most likely number of clusters was *K = *2 (∆*K *= 1,407), which was a similar result to the that obtained with the whole dataset. The locations are: APE (Arabian Peninsula), EUR (Europa, Scattered Islands), DGA (Diego Garcia, Chagos Archipelago), CKE (Cocos(Keeling)), CHR (Christmas), IND (Indonesia), PHI (Philippines) and OKI (Okinawa)

**Figure A6:**
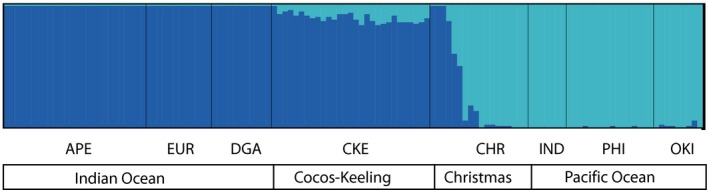
Bayesian cluster analysis performed by structure based in 118 divergent SNP outlier loci of *Dascyllus trimaculatus*. The most likely number of clusters was *K *= 2 (∆*K *= 13,8274), similar to the results with the complete dataset. The locations are: APE (Arabian Peninsula), EUR (Europa, Scattered Islands), DGA (Diego Garcia, Chagos Archipelago), CKE (Cocos(Keeling)), CHR (Christmas), IND (Indonesia), PHI (Philippines) and OKI (Okinawa)

Of the 2,818 loci, we identified 1,949 neutral loci (between the 95% quantiles), 118 divergent loci (high *F*_ST_ outliers, above the 99% quantile), and 289 balancing loci (low *F*_ST_ outliers, below the 1% quantile). A total of 462 loci were unclassified (between the quantiles). structure results with the neutral loci (Figure A5) and the divergent loci (Figure A6) showed similar results than the one with all 2,818 loci. Further, the most likely number of clusters remained as *K* = 2 (Figures A5 and A6), while analyses with the balancing loci showed no population structure.

**Figure 8:**
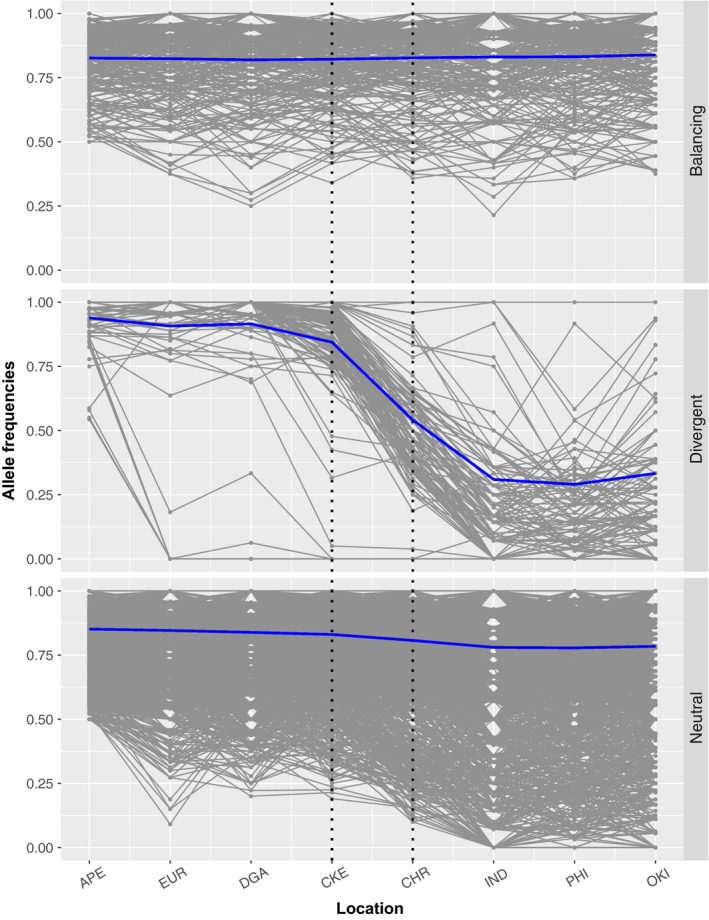
Allele frequencies of loci under balancing (289), divergent (118) and neutral (1949) differentiation. APE: Arabian Peninsula; EUR: Europa, Scattered Islands; DGA: Diego Garcia, Chagos Archipelago; CKE: Cocos (Keeling) Islands; CHR: Christmas Island; IND: Indonesia; PHI: Philippines; OKI: Okinawa. The blue line indicates average allele frequency, and the dashed lines indicate suture zone locations

**Figure A7:**
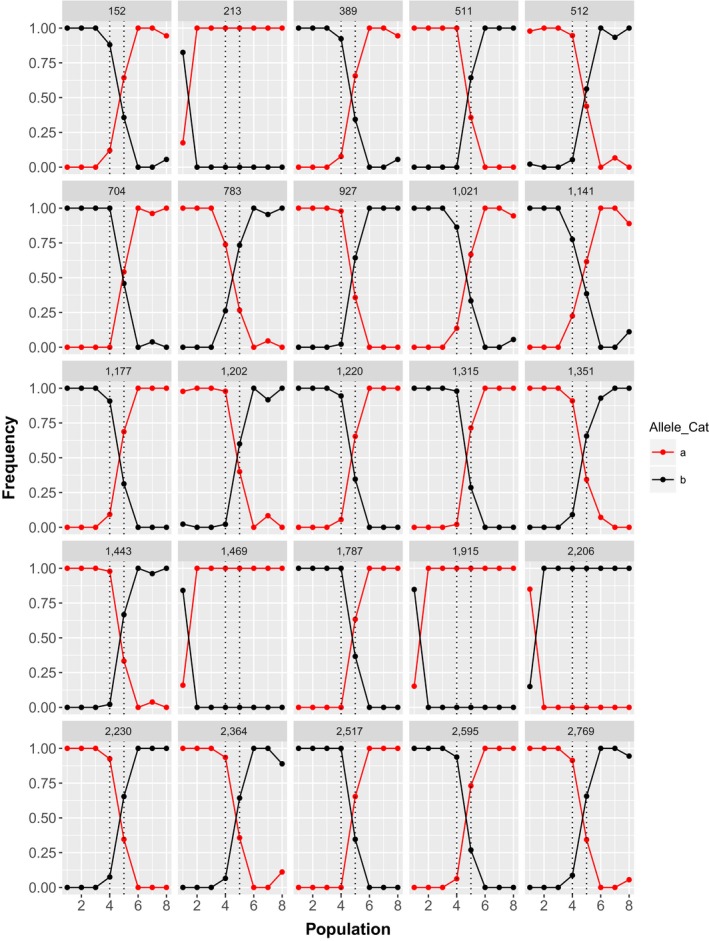
Allele frequencies of a random subsample of 25 loci with the highest Fst (i.e. outliers potentially under divergent selection). The numbers at the top of each graph are the names of the loci. In the Y‐axis, the populations are represented by numbers: Arabian Peninsula (APE, 1); = Europa, Scattered Islands (EUR, 2); Diego Garcia, Chagos Archipelago (DGA, 3); Cocos (Keeling) Islands (CKE, 4); Christmas Island (CHR, 5); Indonesia (IND, 6); Philippines (PHI, 7); Okinawa (OKI, 8). Dashed lines indicate the location of the suture zones

**Figure A8:**
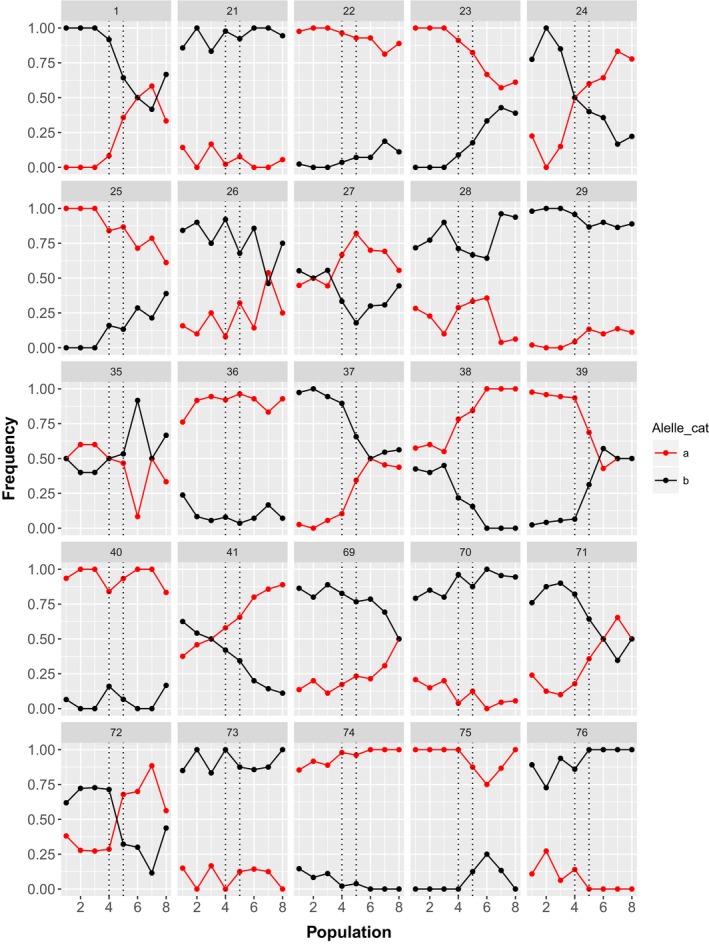
Allele frequencies of a random subsample of 25 neutral loci outliers. The numbers at the top of each graph are the names of the loci. In the *Y*‐axis, the populations are represented by numbers: Arabian Peninsula (APE, 1); = Europa, Scattered Islands (EUR, 2); Diego Garcia, Chagos Archipelago (DGA, 3); Cocos (Keeling) Islands (CKE, 4); Christmas Island (CHR, 5); Indonesia (IND, 6); Philippines (PHI, 7); Okinawa (OKI, 8). Dashed lines indicate the location of the suture zones

**Figure A9:**
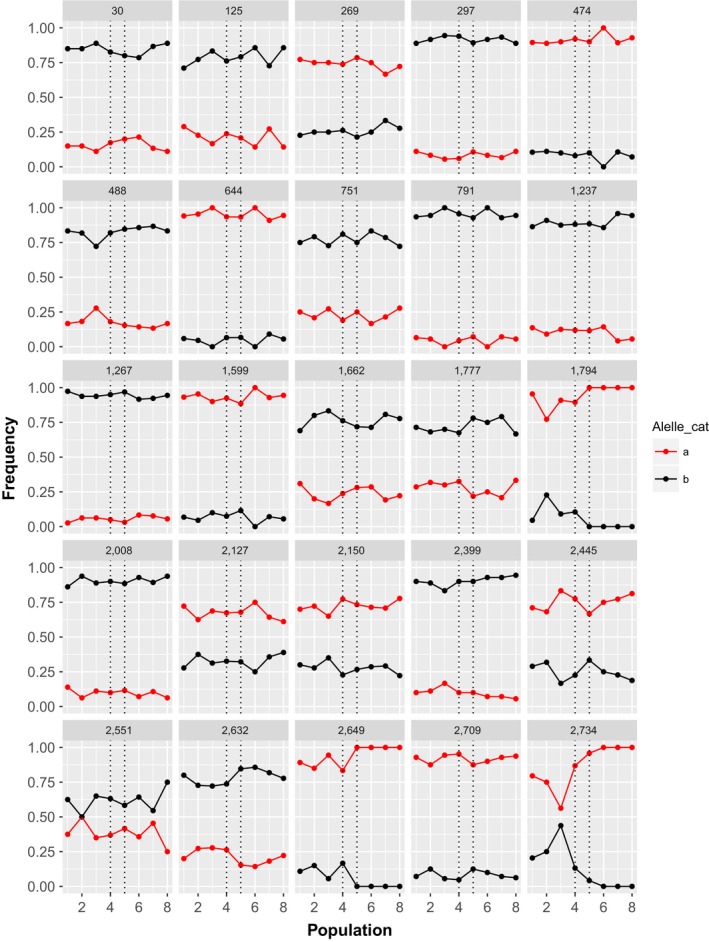
Allele frequencies of a random subsample of 25 loci with the lowest Fst (i.e., outliers potentially under balancing selection). The numbers at the top of each graph are the names of the loci. In the Y‐axis, the populations are represented by numbers: Arabian Peninsula (APE, 1); = Europa, Scattered Islands (EUR, 2); Diego Garcia, Chagos Archipelago (DGA, 3); Cocos (Keeling) Islands (CKE, 4); Christmas Island (CHR, 5); Indonesia (IND, 6); Philippines (PHI, 7); Okinawa (OKI, 8). Dashed lines indicate the location of the suture zones

The graphs of the allele frequencies showed the expected patterns, as the highly divergent loci revealed a steep cline on the suture zone, neutral loci exhibit a less steep transition, and the balancing ones, showed little variation (Figure 8 and Figures A7, A8, A9).

Of the 50 loci that were fixed between Pacific Ocean and Indian Ocean individuals (*F*_ST_ = 1), 17 correspond to protein coding regions (Table A3). Allelic frequencies at these loci varied among populations. By definition, allelic frequency was 100% for alternative alleles in the Indian Ocean and the Pacific. For the suture zone, allelic frequencies were very different. Indeed, the CHR population had a higher allelic frequency for the Pacific alleles (frequency of allele *q* = 0.685, with a narrow range, *q* = 0.750–0.633, Figure 5). In contrast, for the CKE population, allelic frequencies were highest for the Indian Ocean alleles (frequency of allele *p* = 0.925, with also a relatively narrow range of frequencies, *p* = 0.960–0.813, Figure 5). On average, allelic frequency of the most common allele was higher in CKE populations than in CHR populations (*p* = 0.925 vs. *q* = 0.685, Figure 5).

## 4 DISCUSSION

### 4.1 Contrasting patterns of hybridization across taxa

In general, decades of studies in fishes suggest hybridization is facilitated by two components: differential abundance and spawning mode. Indeed, for hybridization to be facilitated, the abundance of hybridizing species is expected to be very different, with one species being abundant and the other being rare (Hubbs, 1955), and when reproduction occurs, broadcast spawning is expected to increase the likelihood of successful hybridization (Nydam & Harrison, 2011). This pattern is observed in the Cocos–Christmas suture zone, as 14 out of the 15 hybrids are broadcast spawners and usually one of the two species involved in the hybridization is rare (Hobbs & Allen, 2014). For substrate spawners, such as *Chromis* and *Dascyllus* (this study), hybridization requires one male to attract a female from a different lineage to his nest. Also, the fate of the eggs in substrate spawners versus broadcast spawners could be different. Broadcast and substrate spawners can have high rates of self‐recruitment (Almany, Berumen, Thorrold, Planes, & Jones, 2007), but they may have different abilities of long‐distance dispersal, as broadcast spawners have been observed to introgress beyond the hybrid zone. This is seen in butterflyfish species that are pair spawners (McMillan, Weigt, & Palumbi, 1999), as well as surgeonfishes that spawn in groups and have very long pelagic larval durations (DiBattista et al., 2016). In addition, the outcomes of hybridization at CHR and CKE could be different for these other species. In these cases, the isolation of Cocos may not be maintained due to differences in dispersal ability.

### 4.2 Contrasting patterns of hybridization across a suture zone

Both our fish visual surveys and genetic data indicate that *Dascyllus trimaculatus* hybridization is common and ongoing at CHR. There is a clear signature of hybrid and backcrossed individuals based on the SNP assignment tests. Further visual surveys show phenotypes from both lineages and intermediates are well represented, and divergent morphs were observed courting and potentially spawning together during the field surveys. Even though color and genetics do not always match, the presence of both morphs and intermediates is indicative of hybridization.

At CHR, the Pacific lineage is more common than the Indian Ocean one, indicating higher connectivity with the Pacific Ocean. The closest location to CHR is Java, Indonesia, at approximately 350 km (James & McAllan, 2014). The prevailing direction of major currents is from the Pacific Ocean to the Indian Ocean, the South Equatorial Current (SEC), and the Indonesian Throughflow (ITF) most likely carried Pacific Ocean larvae to CHR, as they typically move from east to west (Nieblas, Demarcq, Drushka, Sloyan, & Bonhommeau, 2014; Yang et al., 2015). Propagules of Indian Ocean lineages are less likely to arrive at CHR because they must travel a greater distance. The nearest Indian Ocean populations (Northern Indonesia, Myanmar; Sri Lanka; Chagos) are more than 1,000 km from CKE, and CKE is ~900 km from CHR (Hobbs, Newman, et al., 2014). Furthermore, dispersal from most of these locations would be against the prevailing current. However, the South Java Current (SJC) may aid larval dispersal from Northern Indonesia (e.g., Sumatra) to CHR.

Although Christmas Island is relatively small, we observed the highest gene diversity in this population due to the presence of both Pacific and Indian Ocean lineages. In addition, fish from CHR with Pacific mitochondrial haplotypes have higher nucleotide diversity than other samples from the Pacific. Increased genetic diversity is expected in introgressed and hybrid populations, as new genetic material is introduced in the Pacific lineage. It is not possible, however, to determine whether the introgressed alleles are deleterious or advantageous for the population.

Our SNP results suggest that at CKE hybridization could have been common in the recent past, but is currently rare. With the application of genomic analyses, it was possible to detect that most of the samples are backcrosses, as individuals are a mixture of the Indian and Pacific lineages, with ~80% or more of Indian Ocean genotype. However, no F1 individuals were observed, suggesting that hybridization is no longer occurring. SNP analysis could not identify “pure” individuals, suggesting that the populations from CKE are relatively isolated, and the arrival of pure individuals from elsewhere is rare. Historically and based on its geographic position, CKE had stronger connections with the Indian lineage, all individuals had Indian mtDNA haplotypes, and the black fin (Indian) phenotype was the most common one.

It is possible that the three‐spot dascyllus population of CKE is largely relying on self‐recruitment at present, as it is located ~900 km from CHR and more than 1,000 km from Indonesia or any other land‐mass (Hobbs, Newman, et al., 2014). Based on biogeographic observations, it has been suggested that populations of reef fish at CKE are likely maintained by self‐recruitment (Hobbs, Newman, et al., 2014). This idea is also supported by the *Dascyllus trimaculatus* molecular indices, as the gene diversity was the lowest compared to other Indian Ocean locations, and by the DAPC results, as CKE was characterized as a separate cluster. The *D. trimaculatus* depth limit is 55 meters (Allen, 1991); thus, connectivity through the deep neighboring seamounts is unlikely. Thus, to colonize CKE, larvae need to travel large distances (~1,000 km) and settled individuals need to use the scarce available habitats (Hobbs, Newman, et al., 2014). Larvae from elsewhere may not reach the island frequently or die in transit, due to lack of food or complex current reversals surrounding this area (Nieblas et al., 2014). Alternatively, recently settled larvae arrive but only hybrids survive, due to local environmental or ecological conditions. Overall, CKE may be colonized by infrequent vagrants from pure populations, but not enough to maintain a genomic signature of ongoing hybridization or to show genetically pure lineages in our population sample. Self‐recruitment at CKE is likely leading to the formation of a hybrid lineage (as shown in Figure 7) that may continue to evolve into a new species. The evolution of new species from hybridization (hybrid speciation) has been shown for freshwater fishes (Abbott et al., 2013; Meier et al., 2017) and it has been suspected for some anemonefishes (Litsios & Salamin, 2014).

Interestingly, two mitochondrial haplotypes were found in CHR, whereas only one mitochondrial haplotype was found in CKE. The CKE haplotype corresponds to the one in the Indian Ocean lineage. Further, there appears to be mitonuclear discordance as there are, at CHR, individuals with predominant SNPs from the Indian Ocean that have mitochondrial haplotypes from the Pacific (Figure 4). The opposite is also true (i.e., predominant Pacific SNPs with Indian Ocean mitochondria; Figure 4), which further suggests there is rampant hybridization in the suture zone at CHR, but not at CKE. This has been previously reported in marine fishes, where hybridization can lead to different mitonuclear combinations across closely related groups (Bernal, Gaither, Simison, & Rocha, 2017). These events will continue until there is some selective advantage maintaining certain combinations of mitochondrial haplotypes with specific nuclear backgrounds (Hill, 2016), which could already be happening at CKE.

### 4.3 Evolutionary implications

The Cocos–Christmas hybrid zone is a product of secondary contact; the seaway connecting Indian and Pacific populations has opened and closed several times during glacial cycles. Many other sister species with Pleistocene origins overlap at this location (Gaither & Rocha, 2013). Our data do not show any evidence of introgression beyond CHR and CKE due to restricted gene flow. The Sunda Shelf barrier might be strong enough to restrict gene flow between Pacific and Indian Ocean populations, even during high sea level stands, and maintain their differences. Additionally, the Pacific Ocean lineage may be maladapted in the Indian Ocean, and vice versa (Hobbs & Salmond, 2008), making their expansion improbable. Finally, since multiple hybrids and second‐generation backcrossed individuals were found in the suture zone, it is possible that geographic isolation of the two lineages is what maintains the difference.

In addition, sexual selection may play a role in the maintenance of the hybrid zone. Some *Dascyllus* species have UV vision capabilities (Losey, 2003) and there are pomacentrids that have distinctive markings of the face for communication (Siebeck, Parker, Sprenger, Mäthger, & Wallis, 2010). Further, *D. trimaculatus* males produce sounds to attract females, and there are reported differences in the sound period between Indian Ocean and French Polynesian *D. trimaculatus* (Parmentier, Lecchini, Frederich, Brié, & Mann, 2009). However, there are no detailed observations on the frequency of mating preferences in the suture zone, and the observation of mixed color phenotype spawning at CHR shows that there are no strict mate choice preferences related to the color of the fin. Further, there is no evidence that the white/transparent versus dark coloration of the trailing edge of the dorsal fin has any adaptive value or communication function, but may be correlated with other traits.

Another potential explanation for the lack of Pacific Ocean mitochondrial haplotypes in the CKE population, and how the differences between the two lineages are maintained, could be that there are barriers to gene flow due to intrinsic mitochondrial–nuclear genetic incompatibilities. Populations evolving in allopatry tend to fix new alleles involved in Bateson–Dobzhansky–Muller incompatibilities in hybrid genomes which are recognized as a common substrate for speciation (Burton & Barreto, 2012). However, this hypothesis is unlikely because two individuals collected in CHR have Pacific Ocean mitochondrial haplotypes and predominantly Indian Ocean SNP genotypes (Figure 4). The absence of Pacific Ocean mitochondrial haplotypes in CKE may therefore be due to assortative mating rather than nuclear–mitochondrial genetic incompatibility. Indeed, in CHR, where both mitochondrial haplotypes are present, data are consistent with a preference for assortative mating. Out of the four possible mating combinations, zero individuals had an Indian Ocean mtDNA haplotype and a pure Pacific SNP genotype, one individual had a Pacific mtDNA haplotype and a pure Indian Ocean SNP genotype, while the pure matings of Indian Ocean and Pacific mt‐nuclear combinations were found in 2 and 6 individuals, respectively (Figure A1). Such low numbers prevent definitive answers but are indeed suggestive of assortative mating preferences.

### 4.4 Cryptic hybridization

The *D. trimaculatus* complex is a group of species and divergent lineages that lends itself well to studying cryptic hybridization (Leray et al., 2010). Here, we presented the first report of *D. trimaculatus* lineages hybridizing at CHR and CKE. Most of the hybridization cases described before at CHR and CKE highlighted intermediate coloration as the main character to identify hybrids (Hobbs & Allen, 2014) and then applied genetic methods to confirm the hybrid status. This is in stark contrast with our study species, where the slight color differences had not been formally evaluated, and where it is especially hard to detect intermediate morphs. Further, if mitochondrial markers were used exclusively, this elusive case of hybridization would have remained undetected. Hence, this study highlights the importance of using SNPs in detecting hybridization, and should be applied in species where morphological differences are less evident or completely absent. SNPs may also be useful to identify backcrosses of more conspicuous hybrid fishes (like *Chaetodon* and *Centropyge*) and understand the relationship of hybrids and color, which as shown in this study, may not be directly related. Many other Indian–Pacific sister species/lineages co‐occur at CHR and CKE islands (Hobbs & Salmond, 2008) and in areas of Indonesia (Gaither & Rocha, 2013). While hybrid individuals of these have not yet been detected by intermediate coloration, applying genomic tools could reveal additional cases of hybridization.

## 5 CONCLUSIONS

Our research shows that in two locations of a single suture zone, frequency of hybridization is variable, depending on the geographic isolation of the population. CHR populations are currently connected and receiving constant influx from both lineages, with greater influence from the Pacific Ocean. In contrast, CKE populations are geographically isolated and most likely self‐recruiting. The Cocos and Christmas suture zone is a well‐suited system to investigate the effects of the frequency of gene flow on the evolutionary outcomes of hybridization. Future studies should assess the genomic patterns of introgression in multiple species pairs to understand the role of hybridization on the genomic architecture, and if there are changes in the evolutionary trajectory of a whole reef community.

## CONFLICT OF INTEREST

None declared.

## AUTHOR CONTRIBUTIONS

E.S., G.B., J.P.H., M.L.B., and L.A.R. designed the research; E.S. conducted laboratory work; E.S. and G.B. analyzed the genetic data; B.S. contributed to bioinformatics analysis and tools; J.P.H. conducted and analyzed fish surveys; E.S., G.B., M.A.B., M.L.B., J.P.H., and L.A.R. discussed the results and data interpretation. E.S. wrote the manuscript with input from all the authors.

## Data Availability

MtDNA sequences can be found in GenBank (accession numbers MN928258‐MN928378), and Fastq sequences are available at NCBI's SRA (accession numbers: SAMN13326615‐SAMN13326742). Associated metadata is available on the Genomic Observatories MetaDatabase (GeOMe: https://geome-db.org/, Diversity of the IndoPacific Project (DIPnet), expedition GUID: https://n2t.net/ark:/21547/DLu2, Deck et al., 2017).

## APPENDIX A

**Table A1:** Observations of *Dascyllus trimaculatus* color morphs from different locations within the Indian and Pacific Oceans (P = observations in this study, F = photographs from http://www.flickr.com)

Ocean/Sea	Country	Locations	Color
Indian Ocean/Red Sea	Saudi Arabia	Several locations covering North, Central and South coast (P)	B
Indian Ocean/Red Sea	Egypt	South Sinai (F)	B
Indian Ocean/Red Sea	Jordan	Aqaba (F)	B
Indian Ocean	Oman	n.s (F)	B
Indian Ocean	Somaliland	n.s. (P)	B
Indian Ocean	Kenya	n.s (F)	B
Indian Ocean	Madagascar	n.s. (F)	B
Indian Ocean	Maldives	n.s. (F), n.s. (P)	B
Indian Ocean	Thailand	Ko Racha Yai (F)	B
Indian Ocean	Tanzania	Nungwi, Zanzibar North (F)	B
Indian Ocean	Indonesia	Pulau Weh, Sumatra (F)	B
Indian Ocean	Sri Lanka	Trincomalee (F)	B
Indian Ocean	Mauritius	Videos https://www.youtube.com/watch?v=IV1lBh0Tj8c http://www.taxi-inmauritius.com/scuba-diving/	B
Pacific	Solomon Islands	Lola Island (P), Biliki (P), Susuhite (P)	W
Pacific	Guam	n.s. (P)	W
Pacific	Philippines	n.s. (P)	W
Pacific	Fiji	n.s. (P), n.s. (F)	W
Pacific	Micronesia	Pohnpei (P), Kosrae (P), Yap state, various islands (P)	W
Pacific	Taiwan	Dongsha atoll (P)	W
Pacific	Indonesia	Sulawesi (P), Raja Ampat (F), Cenderawashih Bay (F), Sumatra, Pengah Kecik (F)	W
Pacific	Thailand	Surat Thani (F)	W
Pacific	Vanuatu	n.s. (F)	W
Pacific	Japan	Okinawa (F)	W
Pacific	Philippines	n.s. (P), Bagalangit, Calabarzon (F), Cebu (F)	W
Pacific	Palau	Peleliu	W
Pacific	Australia	New South Wales (F), Ningaloo (F)	W

Note

The color section refers to the pattern of the trailing edge of the dorsal fin, that could either be black (B), or white/transparent (W). French Polynesian *D. trimaculatus* also have the trailing edge of the dorsal fin transparent, however this location is not included because this represents a separate genetic lineage (Bernardi et al., 2003).

**Table A2:** Estimates of divergence (*F* _st_) for the SNP dataset of *Dascyllus trimaculatus*

	APE	EUR	DGA	CKE	CHR	IND	PHI	OKI
APE	***							
EUR	**0.0057**	***						
DGA	**0.0101**	−0.0003	***					
CKE	**0.0298**	**0.0208**	**0.0265**	***				
CHR	**0.1303**	**0.1258**	**0.1280**	**0.0815**	***			
IND	**0.2629**	**0.2526**	**0.2479**	**0.1944**	0.0306	***		
PHI	**0.2659**	**0.2615**	**0.2623**	**0.2019**	**0.0452**	**0.0033**	***	
OKI	**0.2557**	**0.2443**	**0.2415**	**0.1870**	0.0272	−0.0027	−0.0006	***

Note

Significant pairwise comparisons after Bonferroni corrections are bolded (*p* < .05).

Abbreviations: APE, Arabian Peninsula; CHR, Christmas; CKE, Cocos(Keeling); DGA, Diego Garcia, Chagos Archipelago; EUR, Europa, Scattered Islands; IND, Indonesia; OKI, Okinawa; PHI, Philippines.

**Table A3:** We identified loci with fixed differences (*F* _st_ = 1) between the Pacific and Indian Ocean lineages

Locus	Description	GenBank accession number
12374	PREDICTED: *Acanthochromis polyacanthus* natriuretic peptide receptor 3 (npr3), mRNA	XM_022216443.1
21317	PREDICTED: *Stegastes partitus* sarcoplasmic/endoplasmic reticulum calcium ATPase 2‐like (LOC103356187), mRNA	XM_008280258.1
26427	PREDICTED: *Pygocentrus nattereri* bolA family member 1 (bola1), transcript variant X5, mRNA	XM_017708735.1
43127	PREDICTED: *Stegastes partitus* nucleoporin 205kDa (nup205), mRNA	XM_008292342.1
51754	PREDICTED: *Lates calcarifer* kinesin family member 18A (kif18a), transcript variant X3, mRNA	XM_018686327.1
54344	PREDICTED: *Mastacembelus armatus* insulin like growth factor 2 mRNA binding protein 3 (igf2bp3), mRNA	XM_026300239.1
57325	PREDICTED: *Manihot esculenta* putative phospholipid:diacylglycerol acyltransferase 2 (LOC110613312), mRNA	XM_021754369.1
81906	PREDICTED: *Carassius auratus* probable global transcription activator SNF2L2 (LOC113079070), transcript variant X6, mRNA	XM_026251300.1
85456	PREDICTED: *Stegastes partitus* transcription regulator protein BACH1‐like (LOC103354492), transcript variant X2, mRNA	XM_008277876.1
93680	PREDICTED: *Denticeps clupeoides* uncharacterized LOC114803462 (LOC114803462), transcript variant X1, ncRNA	XR_003751921.1
95244	PREDICTED: *Gouania willdenowi* transcription factor 7‐like (LOC114459876), transcript variant X1, misc_RNA	XR_003673501.1
99120	PREDICTED: *Amphiprion ocellaris* glucocorticoid induced 1 (glcci1), transcript variant X4, mRNA	XM_023280143.1
102378	PREDICTED: *Perca flavescens* CREB binding protein (crebbp), transcript variant X6, mRNA	XM_028598686.1
113157	PREDICTED: *Cottoperca gobio* toll interacting protein (tollip), mRNA	XM_029462152.1
119248	PREDICTED: *Acanthochromis polyacanthus* cyclin‐dependent kinase 4‐like (LOC110957520), mRNA	XM_022203577.1
135060	PREDICTED: *Lates calcarifer* uncharacterized LOC108886093 (LOC108886093), mRNA	XM_018680728.1
152476	PREDICTED: *Acanthochromis polyacanthus* cytochrome P450 2J2‐like (LOC110971269), mRNA	XM_022221610.1

Note

Out of the 50 loci identified, 17 had matches to protein‐coding sequences in GenBank.
